# From FGFR3 Hyperactivation to Disease-Modifying Therapy in Pediatric Achondroplasia: Molecular Mechanisms, Clinical Evidence, and Emerging Treatments

**DOI:** 10.3390/children13081121

**Published:** 2026-08-21

**Authors:** Rebecca Cristiana Șerban, Andreea Mitut-Veliscu, Alexandra Dumitra, Liana Marica, Cristina Popescu, Andrei Costache, Șerban Teona, Anca-Lelia Riza, Rodica Dirnu, Renata-Maria Varut, Ioana Streață

**Affiliations:** 1 Regional Centre of Medical Genetics Dolj, Emergency County Hospital Craiova, 200642 Craiova, Romania; rebeca.serban@umfcv.ro (R.C.Ș.); alexandra.dumitra@umfcv.ro (A.D.); ioana.streata@umfcv.ro (I.S.); 2 Laboratory of Human Genomics, University of Medicine and Pharmacy of Craiova, 200638 Craiova, Romaniaanca.riza@umfcv.ro (A.-L.R.); 3 Doctoral School, University of Medicine and Pharmacy of Craiova, 200349 Craiova, Romania; liana.marica@umfcv.ro; 4 Discipline of Anatomy, Department of Anatomy, ENT County Hospital Craiova, University of Medicine and Pharmacy, 200349 Craiova, Romania; 5 Department of Biophysics, University of Medicine and Pharmacy of Craiova, 200638 Craiova, Romania; 6 Emergency Clinical County Hospital Craiova, 200642 Craiova, Romania; teona.serban@umfcv.ro; 7 Department of Health and Motricity, Faculty of Medical and Behavioral Sciences, Constantin Brâncuși University of Târgu Jiu, 210185 Târgu Jiu, Romania; rodica.dirnu@e-ucb.ro; 8 Research Methodology Department, Faculty of Pharmacy, University of Medicine and Pharmacy of Craiova, 200349 Craiova, Romania; renata.varut@umfcv.ro

**Keywords:** achondroplasia, FGFR3, growth plate, chondrocytes, endochondral ossification, MAPK/ERK signaling, vosoritide, CNP analogues, precision therapy, emerging treatments

## Abstract

**Highlights:**

**What are the main findings?**

- FGFR3 hyperactivation disrupts growth-plate signaling and endochondral bone growth.
- Vosoritide confirms that achondroplasia can be treated with disease-modifying therapy.

**What are the implications of the main findings?**

- Treatment is shifting toward targeted, mechanism-based therapies.
- Long-term studies are needed to confirm benefits beyond growth velocity.

**Abstract:**

Background/Objectives: Achondroplasia is the most common genetic skeletal dysplasia associated with disproportionate short stature and is primarily caused by gain-of-function variants in the fibroblast growth factor receptor 3 (FGFR3) gene. Constitutive FGFR3 activation disrupts growth plate homeostasis and endochondral ossification through complex alterations in chondrocyte proliferation, differentiation, hypertrophy, extracellular matrix organization, and intracellular signaling. The increasing understanding of these mechanisms has enabled the transition from exclusively supportive management toward disease-modifying and precision-based therapeutic strategies. This narrative review aimed to critically synthesize current evidence on the genetic basis, molecular pathogenesis, growth plate abnormalities, and current and emerging targeted therapies in achondroplasia. Methods: A narrative literature review was conducted using PubMed/MEDLINE, Scopus, and Web of Science Core Collection, with Google Scholar used as a supplementary source, together with manual screening of the reference lists of relevant original studies, clinical trials, reviews, consensus documents, and clinical guidelines. The principal literature search covered publications from January 2010 to March 2026, while selected seminal primary studies published before 2010 were included when necessary to document the original identification of pathogenic FGFR3 variants and foundational mechanisms of FGFR3-mediated growth plate regulation. Particular emphasis was placed on FGFR3 variants, receptor activation mechanisms, growth plate dysfunction, intracellular signaling pathways, vosoritide, C-type natriuretic peptide-based therapies, FGFR3 inhibitors, ligand–receptor blockade, drug repurposing, Wnt/β-catenin modulation, and gene-based therapeutic approaches. Results: Achondroplasia is characterized by marked molecular homogeneity, with the recurrent p.Gly380Arg substitution accounting for most cases. Mutant FGFR3 displays sustained activity through partial ligand independence, enhanced receptor dimerization and kinase activation, increased receptor stability, and reduced degradation. Excessive signaling through MAPK/ERK, STAT, PI3K/AKT, IHH/PTHrP, and related pathways impairs chondrocyte proliferation and hypertrophic differentiation, alters extracellular matrix turnover, disrupts primary cilium function, and reduces longitudinal bone growth. Vosoritide provides clinical proof that pharmacological modulation of FGFR3-related signaling can improve growth velocity. Additional therapeutic strategies under clinical or preclinical investigation include long-acting CNP analogues, selective FGFR inhibitors, decoy receptors, RNA aptamers, repurposed drugs, Wnt/DKK1 pathway modulation, and gene- or enhancer-targeted interventions. Conclusions: Achondroplasia is increasingly understood as a disorder of dysregulated growth plate signaling rather than solely a condition of reduced stature. Although vosoritide has established the feasibility of disease-modifying treatment, substantial uncertainty remains regarding final adult height, skeletal proportionality, cranio-spinal development, orthopedic outcomes, and long-term safety. Future progress will depend on mechanistically informed therapeutic combinations, improved biomarkers, advanced cellular and animal models, and long-term clinical and real-world evidence.

## 1. Introduction

Achondroplasia is the most common skeletal dysplasia associated with disproportionate short stature and the most frequent genetic cause of this phenotype. Its estimated prevalence varies across populations and studies, ranging from approximately 1 in 10,000 to 1 in 30,000 live births, with other estimates reporting 3.72–4.6 cases per 100,000 births [1,2,3]. Although classified as a rare disorder, achondroplasia represents an important model for understanding the molecular regulation of skeletal growth because its genetic basis is highly homogeneous and its pathogenic mechanism directly affects growth plate function.

Achondroplasia is caused by heterozygous gain-of-function variants in the fibroblast growth factor receptor 3 (FGFR3) gene, most commonly the recurrent p.Gly380Arg substitution. The causal relationship between recurrent FGFR3 variants and achondroplasia was established independently in seminal molecular studies published in 1994 and subsequently confirmed in larger mutation series [4,5,6]. Constitutive activation of FGFR3 exaggerates the physiological inhibitory role of this receptor in skeletal development, suppressing chondrocyte proliferation and hypertrophic differentiation and thereby impairing endochondral ossification. Because endochondral bone formation contributes not only to long-bone elongation but also to vertebral, cranial base, and mandibular development, abnormal FGFR3 signaling produces widespread disturbances in skeletal growth and modeling [7,8,9].

The molecular effects of FGFR3 hyperactivation extend beyond a simple reduction in chondrocyte proliferation. Mutant receptor activity alters several interconnected intracellular signaling networks, particularly the RAS–RAF–MEK–ERK and STAT pathways, while additional involvement of PI3K/AKT, IHH/PTHrP, SOX9, and Wnt/β-catenin signaling has also been described [8,9]. These abnormalities affect cell-cycle regulation, chondrocyte maturation, extracellular matrix production and degradation, trabecular bone formation, and primary cilium function. Consequently, achondroplasia is increasingly understood as a complex disorder of growth plate organization and signaling rather than solely as a quantitative defect in longitudinal bone growth.

Recent experimental evidence indicates that modulation of Fgfr3 expression in cartilage can influence the severity of skeletal abnormalities in murine models, suggesting that receptor expression level may act as an additional modifier of the skeletal phenotype [10]. Enhancer elements involved in the cellular regulation of FGFR3 expression, such as the cartilage-associated −29E enhancer, have therefore emerged as potential therapeutic targets. Experimental reduction of Fgfr3 expression through enhancer deletion has improved growth plate architecture, long-bone elongation, vertebral development, and skull base morphology in animal models, suggesting that modulation of receptor expression may represent an alternative to direct inhibition of receptor activity [11].

Historically, the treatment of achondroplasia was predominantly supportive and directed toward established skeletal and functional consequences. Advances in the understanding of FGFR3 biology have, however, enabled a transition toward disease-modifying therapy. Vosoritide, a modified analogue of C-type natriuretic peptide, counteracts FGFR3-dependent MAPK/ERK signaling through activation of the NPR-B/cGMP pathway and has provided clinical proof that growth plate dysfunction in achondroplasia is pharmacologically modifiable [12,13,14,15]. Its development has established a therapeutic framework for targeting the molecular mechanisms responsible for impaired endochondral growth.

Vosoritide does not correct the pathogenic FGFR3 variant and counteracts only selected downstream consequences of receptor hyperactivation. Following the identification of activating FGFR3 variants and the progressive characterization of the associated signaling disturbances, several therapeutic approaches acting at different molecular levels have been considered or developed. These include long-acting CNP analogues, selective FGFR tyrosine kinase inhibitors, soluble decoy receptors, RNA aptamers that interfere with ligand–receptor interactions, repurposed pharmacological agents, Wnt/DKK1 pathway modulators, and experimental gene-, RNA-, or enhancer-based interventions [9,10,11,12,16].

Despite rapid therapeutic progress, several questions remain unresolved. The relative contribution of individual FGFR3-dependent pathways to growth plate dysfunction has not been fully defined, and it remains uncertain whether molecular therapies can modify skeletal architecture, body proportionality, cranio-spinal development, limb deformities, and final adult height in addition to increasing growth velocity. The optimal timing, duration, sequencing, and potential combination of therapies also require further investigation.

Therefore, this narrative review aims to critically synthesize current evidence regarding the genetic and molecular basis of achondroplasia, the mechanisms through which FGFR3 hyperactivation disrupts growth plate homeostasis and endochondral ossification, and the current and emerging therapeutic strategies designed to modify the underlying disease process. Particular emphasis is placed on FGFR3-dependent signaling networks, growth plate cellular abnormalities, vosoritide and other CNP-based therapies, direct receptor inhibition, ligand–receptor blockade, alternative molecular targets, and gene- or enhancer-based approaches.

From a pediatric perspective, therapeutic benefit cannot be defined by linear growth alone. Infancy and early childhood represent periods of particular vulnerability to foramen magnum stenosis, cervicomedullary compression, sleep-disordered breathing, delayed motor development, and progressive skeletal disproportion [17,18,19]. During later childhood and adolescence, additional concerns include lower-limb malalignment, spinal canal narrowing, pain, fatigue, restricted physical functioning, psychosocial burden, and the potential need for orthopedic intervention [20,21]. For children receiving long-term pharmacological therapy, treatment fatigue, treatment burden, and adherence represent additional therapy-related considerations [22,23,24]. Therefore, the evaluation of disease-modifying therapies should incorporate age-specific structural, neurological, respiratory, functional, safety, and patient-reported outcomes in addition to annualized growth velocity [24,25,26,27]. Accordingly, this review not only examines how emerging therapies modify FGFR3-related signaling but also critically considers the extent to which available pediatric evidence supports clinically meaningful benefits beyond height gain. This distinction is essential when translating molecular efficacy into long-term therapeutic value for children and their families.

## 2. Materials and Methods

### 2.1. Literature Search Strategy

This narrative review was designed to provide an updated and mechanistically oriented synthesis of the genetic basis, molecular pathogenesis, growth plate abnormalities, and current and emerging targeted therapies in achondroplasia. A comprehensive literature search was conducted using PubMed/MEDLINE, Scopus, and Web of Science Core Collection. Google Scholar was used as a supplementary source to identify additional relevant publications and for citation tracking. The electronic search was complemented by manual screening of the reference lists of selected original articles, clinical trials, review papers, consensus documents, and clinical guidelines.

The principal literature search focused on publications from January 2010 to March 2026. However, a lower publication-date restriction was applied to a limited number of seminal primary studies considered essential for establishing the genetic basis of achondroplasia and the fundamental biological role of FGFR3 in growth plate regulation and endochondral ossification. These earlier studies were identified through backward citation searching and were included only when they provided foundational evidence that could not be appropriately represented solely by more recent secondary literature. Search terms were used individually and in different combinations and included “achondroplasia”, “FGFR3”, “FGFR3 mutation”, “p.Gly380Arg”, “growth plate”, “growth plate chondrocytes”, “chondrocyte proliferation”, “chondrocyte hypertrophy”, “endochondral ossification”, “MAPK/ERK”, “RAS/MAPK”, “STAT signaling”, “PI3K/AKT”, “IHH/PTHrP”, “SOX9”, “Wnt/β-catenin”, “DKK1”, “extracellular matrix”, “primary cilium”, “vosoritide”, “C-type natriuretic peptide”, “NPR-B”, “TransCon CNP”, “infigratinib”, “recifercept”, “RBM-007”, “meclizine”, “FGFR inhibition”, “drug repurposing”, “gene therapy”, “CRISPR-Cas9”, “RNA interference”, and “FGFR3 enhancer”.

### 2.2. Eligibility and Selection Criteria

Publications were considered eligible when they addressed at least one of the principal topics of the review: the genetic basis of achondroplasia, molecular mechanisms of FGFR3 activation, intracellular signaling pathways, growth plate and chondrocyte dysfunction, impaired endochondral ossification, or pharmacological and genetic strategies targeting the underlying disease mechanism. The review considered original experimental studies, in vitro and in vivo investigations, translational studies, clinical trials, long-term extension studies, consensus statements, clinical guidelines, systematic reviews, and high-quality narrative reviews published in English. Studies involving pediatric populations were included when they provided relevant information regarding the pharmacological efficacy, safety, biological effects, or clinical implementation of disease-modifying therapies. Priority was given to original mechanistic studies, controlled clinical trials, long-term therapeutic studies, recent high-quality reviews, and publications that introduced or evaluated novel molecular targets. Studies published before January 2010 were eligible only when they represented seminal primary contributions establishing the genetic basis of achondroplasia or fundamental mechanisms of FGFR3-mediated growth plate regulation and endochondral ossification. These studies were treated as predefined historical exceptions to the principal publication period and were explicitly cited in the relevant mechanistic sections. Studies addressing clinical complications, surveillance, physical functioning, treatment burden, psychosocial outcomes, health-related quality of life, and child- or caregiver-reported outcomes were included when they informed the pediatric relevance, clinical implementation, or broader value of disease-modifying therapy. Particular attention was given to studies evaluating outcomes beyond linear growth, including body proportionality, limb alignment, cranio-cervical development, respiratory complications, orthopedic interventions, pain, mobility, daily functioning, and treatment adherence.

Adult-only studies were retained only when they provided essential long-term natural-history information or outcomes not yet available in pediatric cohorts. Conference abstracts without sufficient methodological information, duplicate publications, inaccessible full-text reports, and studies without direct relevance to FGFR3 biology, pediatric disease expression, or disease-modifying treatment were excluded.

### 2.3. Evidence Evaluation and Narrative Synthesis

Potentially relevant publications were evaluated according to their scientific relevance, methodological quality, recency, and contribution to the mechanistic or therapeutic understanding of achondroplasia. The selected evidence was organized according to the main levels of disease biology and therapeutic intervention: FGFR3 variants and receptor regulation, mechanisms of FGFR3 hyperactivation, intracellular signaling networks, growth plate cellular abnormalities, skeletal developmental consequences, clinically available disease-modifying therapy, and emerging pharmacological or gene-based approaches. Findings from experimental studies were interpreted separately from evidence derived from clinical trials and routine clinical practice. Particular attention was given to the level of therapeutic development, distinguishing between approved treatment, clinical-stage investigational therapies, and preclinical or experimental strategies. Areas of uncertainty, inconsistent findings, and limitations in the available long-term evidence were also identified and critically discussed. For therapeutic evidence, official approval documents issued by health authorities and peer-reviewed randomized controlled trials were prioritized, followed by long-term extension studies, prospective observational cohorts, clinical registries, and preclinical investigations. Cross-trial comparisons were interpreted cautiously because of differences in age distribution, eligibility criteria, baseline growth assessment, outcome definitions, concomitant therapies, and follow-up duration. Particular attention was given to whether reported benefits were limited to annualized growth velocity or extended to body proportionality, skeletal alignment, physical function, quality of life, and complication-related outcomes.

A narrative review design was selected to integrate heterogeneous molecular, translational, clinical, and patient-centered evidence rather than to estimate a pooled treatment effect. Evidence was therefore synthesized qualitatively without a PRISMA-based study selection process, quantitative meta-analysis, or standardized risk-of-bias assessment, using a pediatric mechanism-to-outcome framework linking therapeutic targets with developmental timing and clinically relevant outcomes.

## 3. Genetic and Molecular Basis

### 3.1. FGFR3 Gene

Achondroplasia is caused by activating missense mutations in the fibroblast growth factor receptor 3 (FGFR3) gene and follows an autosomal dominant pattern of inheritance [3,28]. FGFR3 is located on chromosome 4p16.3 and encodes a transmembrane receptor tyrosine kinase with a major role in skeletal growth, especially in tissues that develop through endochondral ossification [4,7,9]. In normal skeletal development, FGFR3 functions as a physiological brake on bone elongation. It is expressed mainly in growth plate chondrocytes, where it limits both proliferation and hypertrophic differentiation. Its expression is not confined to cartilage, however; FGFR3 has also been identified in condensed mesenchyme, mature osteoblasts, osteoclasts, osteocytes, and selected cranial and intervertebral structures. This broader distribution helps explain why abnormal FGFR3 signaling affects more than long-bone growth alone. In achondroplasia, the receptor’s normal inhibitory role is amplified, shifting it from a regulator of growth into a suppressor of skeletal elongation [7,8,9].

From a molecular standpoint, achondroplasia is unusually homogeneous. Approximately 99% of individuals with achondroplasia carry one of two recurrent FGFR3 variants: c.1138G>A, identified in approximately 98% of cases, or c.1138G>C, identified in approximately 1%. Both variants result in the p.Gly380Arg substitution [4,5,29]. Rare variants, including p.Ser217Cys, p.Gly375Cys, and p.Thr394Ser, have also been reported, showing that the mutational spectrum is not limited to the classic recurrent mutation, even though these alternatives remain uncommon [9,29].

Most achondroplasia-associated variants are located in the transmembrane domain of the receptor, although rare pathogenic substitutions may occur elsewhere in the protein [29]. This distribution is consistent with a gain-of-function mechanism in which pathogenic FGFR3 variants enhance receptor signaling activity and thereby amplify its physiological inhibitory effect on endochondral bone growth. The level of FGFR3 expression may also influence the biological effects of receptor hyperactivation. Recent experimental work suggests that cartilage-specific expression of Fgfr3 is influenced not only by the gene promoter but also by enhancer elements involved in the cellular regulation of gene expression. One such enhancer, located approximately 29 kb upstream of the gene and referred to as −29E, appears to contribute substantially to Fgfr3 expression in cartilage [11]. This finding adds an additional layer to the molecular model of achondroplasia, suggesting that disease severity may depend not only on the coding mutation itself but also on the level of receptor expression in relevant skeletal tissues.

The centrality of FGFR3 has practical consequences for diagnosis and treatment. Because most cases are caused by a small number of recurrent variants, molecular confirmation is now a key part of clinical evaluation, especially when treatment decisions require genetic verification. In some healthcare systems, for example, vosoritide initiation depends on documented confirmation of the Gly380Arg mutation in FGFR3 [14]. Thus, FGFR3 sits at the intersection of pathogenesis, diagnosis, and modern therapeutic decision-making. Achondroplasia follows an autosomal dominant inheritance pattern with essentially complete penetrance. Approximately 80% of cases arise de novo, whereas the remaining cases are inherited from an affected parent; de novo FGFR3 variants are also associated with advanced paternal age [14,16,28]. For genetic counseling, when one parent has heterozygous achondroplasia, each pregnancy carries a 50% probability of inheriting the condition. If both parents are affected, each pregnancy has a 25% probability of average stature, a 50% probability of heterozygous achondroplasia, and a 25% probability of homozygous achondroplasia, which is usually life-limiting. For unaffected parents of a child with an apparently de novo variant, the recurrence risk is low but remains above the population risk because of possible germline mosaicism.

### 3.2. Recurrent FGFR3 Variants

The molecular basis of achondroplasia is a gain-of-function alteration of FGFR3 that results in sustained receptor activation and excessive downstream signaling [7,9]. This enhanced signaling amplifies the physiological inhibitory effect of FGFR3 on growth plate chondrocytes, thereby reducing chondrocyte proliferation and hypertrophic differentiation and impairing endochondral bone growth [9,29].

In most patients, this effect is produced by the recurrent p.Gly380Arg substitution. The variant makes FGFR3 partly ligand-independent and more likely to become activated even without normal fibroblast growth factor stimulation. As a result, signaling remains persistently increased, and chondrocyte proliferation and maturation are chronically suppressed. Experimental studies suggest that this overactivation is not caused by a single event. The mutation appears to favor receptor dimerization, increase tyrosine kinase activity, and stabilize the receptor, which prolongs intracellular signaling. Rare FGFR3 variants seem to reach the same pathogenic endpoint through slightly different mechanisms. Variants such as p.Ser217Cys, p.Ser279Cys, and p.Ser344Cys introduce an additional cysteine residue into the receptor structure, favoring abnormal activation and continuous signaling. Mutant FGFR3 may also undergo reduced lysosomal degradation, allowing the receptor to persist longer within the cell and amplify its biological effect. Although these variants are uncommon, they illustrate an important principle: different structural changes in FGFR3 can converge toward excessive receptor activity and impaired endochondral growth [9,29].

Downstream, FGFR3 overactivation interferes with several signaling pathways. The most consistently implicated are MAPK/ERK, PI3K/AKT, STAT, and RAS/MAPK, all of which participate in the abnormal regulation of chondrocyte behavior in achondroplasia. More recent work also points to secondary disruption of Wnt/β-catenin signaling, suggesting that the molecular consequences of FGFR3 hyperactivation extend beyond the classical growth plate pathways [8,9,16]. This broader signaling disturbance helps explain why achondroplasia is not simply a disorder of reduced linear growth but a complex alteration of cartilage development and skeletal modeling. At the tissue level, the consequences are clear: reduced chondrocyte proliferation, impaired hypertrophic differentiation, and abnormal trabecular bone formation [7,8,9]. Because FGFR3 normally acts as a negative regulator of skeletal growth, its constitutive activation exaggerates this inhibitory role and shifts growth plate dynamics toward reduced longitudinal elongation.

### 3.3. Genotype-Phenotype Correlations

Genotype-phenotype correlations in achondroplasia are unusual when compared with many other skeletal dysplasias, mainly because the disorder is highly homogeneous at the molecular level. Most patients carry the recurrent p.Gly380Arg variant, caused by c.1138G>A in approximately 98–99% of cases and by c.1138G>C in a small minority. This marked molecular homogeneity helps explain why genetic confirmation is usually straightforward and why the phenotype is often clinically recognizable.

The classic achondroplasia phenotype is closely linked to this recurrent mutation. Disproportionate short stature, rhizomelic limb shortening, macrocephaly, frontal bossing, and characteristic skeletal abnormalities are all strongly associated with p.Gly380Arg. This relative molecular homogeneity distinguishes achondroplasia from skeletal dysplasias with broader genetic heterogeneity and more variable clinical expression. In practice, when the phenotype is typical, genetic testing often confirms an already strong clinical suspicion rather than initiating a broad diagnostic investigation [6,29]. This does not mean that genotype–phenotype relationships are completely fixed. Rare FGFR3 variants, including Ser217Cys, Ser279Cys, and Ser344Cys, suggest that different mutations may alter the intensity of receptor activation and, consequently, influence clinical severity [9,29]. Even when the phenotype remains within the achondroplasia spectrum, these variants show that not all activating FGFR3 mutations behave identically. The clearest examples of increased severity come from rare double or compound mutations, where the classic G380R variant occurs together with an additional FGFR3 alteration. These cases have been associated with respiratory distress, pulmonary hypoplasia, hydrocephalus, and cervicomedullary compression [29]. Such observations suggest that phenotype depends not only on the presence of an FGFR3 mutation but also on the degree and complexity of receptor dysregulation.

Experimental work adds another layer to this relationship. In a murine model of achondroplasia, reducing Fgfr3 expression through deletion of the −29E enhancer improved the skeletal phenotype even though the pathogenic mutation itself remained present [11]. This is an important concept: phenotypic severity may reflect both the qualitative effect of the mutation and the quantitative level of FGFR3 expression in cartilage. Thus, achondroplasia is genetically homogeneous in most patients, but not biologically uniform in every respect. The predominance of p.Gly380Arg explains the typical phenotype and the relative simplicity of diagnosis, while rare variants, compound mutations, and differences in receptor expression help account for variation in severity. These nuances may become increasingly relevant as prognosis and treatment move toward more targeted approaches (Table 1). Prenatal suspicion of achondroplasia usually arises during the late second or third trimester, because long-bone shortening may not be apparent at the routine mid-trimester examination. Suggestive findings include progressive rhizomelic shortening, femoral length below the third to fifth percentile, relative macrocephaly, frontal bossing, a depressed nasal bridge, short hands, and characteristic proximal femoral metaphyseal morphology. A cloverleaf skull is not typical of heterozygous achondroplasia and should prompt consideration of other FGFR3-related skeletal dysplasias, particularly thanatophoric dysplasia.

Targeted prenatal FGFR3 testing using chorionic villus sampling or amniocentesis may be offered when ultrasonographic findings suggest achondroplasia, when one or both parents are affected, or when parents of average stature have previously had an affected child.

## 4. Pathophysiology

### 4.1. Endochondral Ossification and Growth Plate Dysfunction

Achondroplasia is primarily a disorder of endochondral ossification, the process responsible for most longitudinal bone growth. Normal skeletal development requires the coordinated progression of growth plate chondrocytes through resting, proliferative, pre-hypertrophic, and hypertrophic stages, followed by extracellular matrix remodeling, vascular invasion, and replacement of cartilage by bone [7,8,9]. In achondroplasia, excessive FGFR3 signaling disrupts this coordinated process, impairing chondrocyte proliferation and progression toward hypertrophic differentiation. At the growth plate level, these molecular abnormalities result in reduced proliferative and hypertrophic zones, disturbed columnar organization, and impaired chondrocyte maturation [8,9]. Excessive FGFR3 signaling also affects cell-cycle control, with increased expression of inhibitors such as p16, p19, p21, and p27 and reduced telomerase activity, while pathways involved in chondrocyte differentiation, including IHH/PTHrP and SOX9-related signaling, are also disturbed [8,9]. Consequently, growth plate architecture and cellular progression are altered, reducing the efficiency of endochondral bone formation. Extracellular matrix homeostasis is affected in parallel. Reduced synthesis of aggrecan and type II collagen, together with increased expression of matrix-degrading enzymes including MMP3, MMP9, MMP10, and MMP13, contributes to disruption of the structural environment required for normal chondrocyte proliferation and maturation [9]. Experimental data have also identified abnormalities of primary-cilium biology, including shortened cilia, altered IFT20 trafficking, and disturbed IHH signaling, further supporting the concept that achondroplasia involves broader alterations of cellular organization and signaling within the growth plate [9].

**Figure 1:**
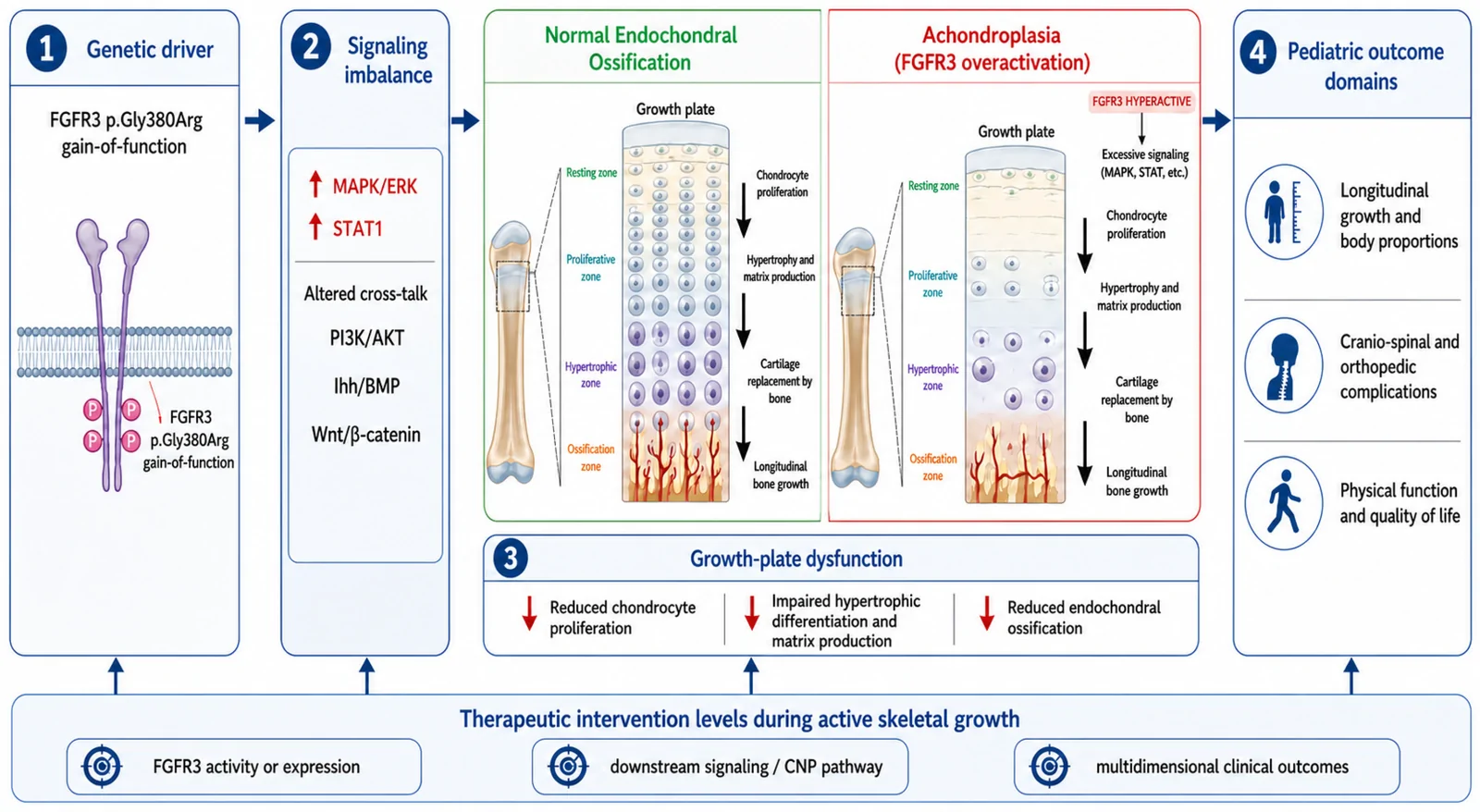
Integrated pediatric FGFR3 mechanism-to-outcome framework in achondroplasia. Recurrent gain-of-function FGFR3 variants cause sustained receptor activation and downstream signaling imbalance, including increased MAPK/ERK and STAT1 activity and altered cross-talk with PI3K/AKT, Ihh/BMP, and Wnt/β-catenin pathways. These alterations reduce chondrocyte proliferation, impair hypertrophic differentiation and matrix production, and compromise endochondral ossification. The framework links growth-plate dysfunction to longitudinal growth and body proportions, cranio-spinal and orthopedic complications, physical function, and quality of life, while indicating general levels of therapeutic intervention during active skeletal growth. Pathway interactions are simplified for clarity. The original illustrations were created with BioRender.com; the integrated framework was redesigned and scientifically verified by the authors and is based on references [7,8,9,10,11,16].

Murine models of achondroplasia support these cellular observations. Reduced Fgfr3 expression has been associated with improved growth plate organization, increased chondrocyte proliferation and hypertrophic differentiation, and improved skeletal growth [11]. These experimental findings demonstrate that sustained modulation of excessive FGFR3 activity or expression can improve growth plate function during active skeletal growth. The therapeutic relevance of the growth plate is also supported clinically by vosoritide, which counteracts downstream FGFR3 signaling through activation of natriuretic peptide receptor B and promotes chondrocyte proliferation and differentiation [12,13]. To integrate these molecular and cellular findings with clinically relevant pediatric consequences, Figure 1 presents a mechanism-to-outcome framework linking FGFR3 gain-of-function to downstream signaling imbalance, growth-plate dysfunction, and multidimensional pediatric outcomes, while indicating the principal levels of therapeutic intervention during active skeletal growth.

### 4.2. Effects on Skeletal Development

The defect in endochondral ossification that defines achondroplasia affects the skeleton as a whole, not only linear growth. Normal skeletal elongation depends on the coordinated proliferation, maturation, and hypertrophy of growth plate chondrocytes. When FGFR3 signaling remains persistently increased, this coordination is disrupted, producing the disproportionate short stature characteristic of the condition [9,19].

The most visible consequence is shortening of the long bones, especially in the proximal limb segments. Experimental models consistently show shortened long bones together with abnormal growth plate organization, supporting the direct link between altered chondrocyte behavior and impaired skeletal elongation [7,8,9]. Longitudinal growth also depends on coordinated development of the perichondrium and periosteum, and these structures may also be affected by abnormal FGFR3 activity. This helps explain why the skeletal phenotype is more complex than simple limb shortening [9,20]. The axial skeleton is also involved. FGFR3 participates in the development of the vertebral column, cranial base, and other skeletal structures, which accounts for vertebral shortening, spinal canal narrowing, and craniofacial abnormalities in achondroplasia. Experimental models have shown shortened vertebral bodies, spinal stenosis, dome-shaped skull deformities, and changes in mature bone architecture, including reduced cortical thickness, altered trabecular bone, and osteopenia [9,11]. These findings suggest that the disease affects both early skeletal growth and later skeletal structure.

One of the most clinically relevant examples is the foramen magnum. In infants with achondroplasia, it is smaller than in unaffected children, partly because of restricted early postnatal growth and premature closure of skull base synchondroses. This can produce a narrowed and malformed cranio-cervical opening, with potential compression of the brainstem and upper cervical spinal cord [18,19,30]. Since the foramen magnum normally enlarges rapidly during the first months of life, impaired growth in this region creates a period of particular neurological vulnerability in infancy [18,19]. Craniofacial development is affected beyond the skull base. Meckel’s cartilage and condylar cartilage contribute to embryonic and postnatal mandibular growth, respectively [31,32]. In achondroplasia, abnormalities in these cartilaginous structures have been associated with mandibular hypoplasia, micrognathia, and altered mandibular shape, with functional as well as aesthetic consequences [31,32]. Recent findings suggest that these changes may involve not only FGFR3 signaling itself but also secondary pathway disturbances, including altered Wnt/β-catenin signaling through increased DKK1 expression [16].

The severity of skeletal involvement may also depend on the level of receptor activity in developing tissues. In murine models, reducing Fgfr3 expression improves long bone growth, vertebral development, skull morphology, and foramen magnum size [11]. This supports the idea that the phenotype is shaped not only by the presence of the pathogenic mutation but also by the degree of FGFR3 signaling. Achondroplasia therefore produces a broad disturbance of skeletal growth and modeling. The long bones, vertebral column, cranial base, foramen magnum, and mandible are all involved, which explains why the condition cannot be reduced to impaired stature alone.

## 5. Current and Emerging Therapeutic Strategies

### 5.1. From Supportive Management to Disease-Modifying Therapy

Before the development of molecularly targeted therapies, the management of achondroplasia was predominantly supportive and directed toward the prevention or correction of established complications. Surveillance, rehabilitation, orthopedic and neurosurgical procedures, respiratory interventions, and environmental adaptations remain essential components of care; however, these approaches do not directly modify the excessive FGFR3 signaling responsible for impaired endochondral ossification [18,20,28,33].

Conventional growth-promoting strategies have also shown limited ability to alter the underlying disease process. Growth hormone may transiently increase growth velocity, particularly during the initial treatment period, but its effect on final adult height and skeletal proportionality remains modest and inconsistent. Similarly, limb-lengthening procedures can increase stature and correct selected deformities, but they are associated with substantial treatment burden and potential complications and do not address the molecular cause of achondroplasia [9,18,20].

The identification of constitutive FGFR3 activation as the central pathogenic mechanism of achondroplasia established the basis for a fundamentally different therapeutic approach. Rather than treating only the structural and functional consequences of the disorder, disease-modifying therapies aim to restore growth plate homeostasis by attenuating excessive receptor signaling, activating antagonistic pathways, preventing ligand–receptor interactions, or reducing FGFR3 expression. Vosoritide represents the first clinically established example of this strategy and provides proof of concept that growth plate dysfunction in achondroplasia is pharmacologically modifiable.

Conventional and molecularly targeted treatments should therefore be considered complementary rather than mutually exclusive. Supportive and surgical interventions remain necessary for established complications, whereas disease-modifying therapies aim to influence the biological mechanisms that generate skeletal abnormalities. Whether early molecular treatment can reduce the incidence or severity of future orthopedic and cranio-spinal complications remains uncertain and requires long-term prospective investigation [18,20,33].

### 5.2. Vosoritide

Vosoritide is the first disease-specific pharmacological therapy approved to increase growth velocity in children with achondroplasia who still have open growth plates. It is a modified recombinant analogue of C-type natriuretic peptide, designed to resist rapid degradation and allow once-daily subcutaneous administration [12,13,14]. Its mechanism of action is based on activation of the CNP/NPR-B pathway. This increases intracellular cGMP, activates PKG2, and inhibits downstream FGFR3 signaling, particularly through the MAPK/ERK cascade [9,10,11,12]. Vosoritide therefore does not correct the FGFR3 mutation itself, but counteracts one of its main biological consequences: excessive inhibition of chondrocyte proliferation and differentiation. Clinical trials have shown a consistent increase in annualized growth velocity. In the pivotal phase 3 study, vosoritide produced an additional gain of approximately 1.57 cm/year compared with placebo, with supportive findings from open-label extension studies [12,13,14,15]. Some analyses estimate an additional height gain of about 3.5 cm after two years compared with untreated children, suggesting that the effect is sustained rather than temporary [15]. Longer follow-up, including data from up to seven years from phase 2 cohorts, has shown continued benefit for height and some improvement in body proportions, without evidence of abnormal acceleration of skeletal maturation.

Available data suggest benefit in children aged 2–5 years as well as in those older than 5 years, while preliminary findings in infants point toward possible improvement in growth and proportionality at even younger ages [12,15]. These infant data remain limited and need confirmation in larger cohorts. Regulatory indications have also changed over time, with approval initially granted for older children and later extended to younger age groups in several regions [12,14]. Response varies between patients. Some children show substantial gains, while others respond more modestly [12]. For this reason, treatment requires long-term follow-up that includes height, weight, growth velocity, body proportions, and continued surveillance for achondroplasia-related complications [14,27].

From a practical point of view, treatment requires molecular confirmation of the diagnosis and evidence that growth plates remain open. In the Australian guidance, continuation depends on the absence of physeal closure, shown either radiographically or indirectly through an annual growth velocity greater than 1.5 cm/year over at least six months. Treatment should be stopped when epiphyseal closure is documented or when growth velocity falls below this threshold in late puberty on two consecutive assessments. These criteria reflect a basic biological limitation: vosoritide can act only while functional growth plate cartilage is still present. The safety profile is generally acceptable in both clinical trials and post-marketing experience. Injection-site reactions and episodes of hypotension are the most frequently reported adverse events and are usually mild and self-limited [14]. In everyday practice, however, the burden of daily injections should not be underestimated, especially in younger children. Discomfort, treatment fatigue, and long-term adherence can all influence the real-world value of therapy [13,14]. These issues should be discussed openly with families before treatment begins. Important uncertainties remain. Although vosoritide improves growth velocity and some body proportions, its effect on final adult height, limb deformities, future orthopedic surgery, pain, quality of life, and cranio-cervical or spinal complications is still unclear [12,18,20]. There is growing interest in whether very early initiation might influence foramen magnum stenosis or obstructive sleep apnea, but this has not yet been demonstrated [17,19,34].

The relationship between vosoritide and orthopedic treatment is another developing area. A pediatric clinical-surgical series suggested that vosoritide could often be continued during limb surgery without major new safety signals or clear deviation from the expected orthopedic course. Reported adverse events were mainly local and transient, while surgical complications were consistent with those expected from orthopedic procedures. The data are still limited, and the optimal timing of pharmacological treatment in relation to orthopedic surgery remains uncertain. Vosoritide has changed the treatment landscape for achondroplasia by targeting a central mechanism of impaired endochondral growth. Its benefit is real, but partial. It should be viewed as part of multidisciplinary care, not as a substitute for surveillance or for conventional management of orthopedic, neurological, respiratory, and functional complications [18,20,33].

From a pediatric clinical perspective, response assessment should extend beyond standing height and annualized growth velocity. Longitudinal monitoring should include upper-to-lower body segment ratio, limb alignment, head circumference, neurological and respiratory status, developmental and functional outcomes, pain, treatment adherence, and child- or caregiver-reported quality of life. At present, evidence that vosoritide prevents cervicomedullary compression, reduces the future need for orthopedic surgery, or substantially improves final adult height remains insufficient. Treatment decisions should therefore be individualized through shared decision-making and should not replace established multidisciplinary surveillance.

### 5.3. Emerging Therapies

Although vosoritide established the feasibility of disease-modifying treatment, the therapeutic landscape has continued to evolve rapidly. One of the most important developments is navepegritide, previously known as TransCon CNP, a long-acting CNP prodrug designed for once-weekly subcutaneous administration. In the randomized, double-blind, placebo-controlled APPROACH trial, 84 children aged 2–11 years received navepegritide or placebo for 52 weeks. Navepegritide produced a least-squares mean treatment difference in annualized growth velocity of 1.49 cm/year compared with placebo. Improvements were also reported in selected measures of lower-limb alignment, including tibial–femoral angle, mechanical axis deviation, and fibula-to-tibia length ratio, together with a favorable change in physical functioning among children younger than five years [35]. In February 2026, navepegritide received accelerated approval from the United States Food and Drug Administration under the trade name Yuviwel for children aged two years and older with achondroplasia and open epiphyses. The approval was based on improvement in annualized growth velocity, while confirmation of benefit on final adult height remains required. Navepegritide should therefore be classified as an approved disease-modifying therapy in the United States rather than as an investigational treatment [36].

Direct inhibition of FGFR signaling has also reached late-stage clinical evaluation. Infigratinib is an orally administered FGFR1–3 tyrosine kinase inhibitor that directly reduces excessive receptor-mediated signaling. In the phase 3 PROPEL 3 trial, 114 children aged 3–17 years were randomly assigned to receive once-daily infigratinib or placebo for 52 weeks. The least-squares mean treatment difference in the change from baseline in annualized height velocity was 1.74 cm/year in favor of infigratinib. Height z-score also improved, whereas the difference in upper-to-lower body segment ratio was not statistically significant in the overall study population. Adverse-event frequencies were similar between the treatment and placebo groups, and no serious adverse events or treatment discontinuations were considered related to the study intervention. These results provide strong clinical support for direct FGFR inhibition but do not yet establish effects on final adult height, spinal and cranio-cervical development, orthopedic burden, or other long-term complications. Infigratinib therefore remains an investigational therapy despite the positive phase 3 results [9,12,37]. Dabogratinib (TYRA-300) is a once-daily oral FGFR3-selective inhibitor currently being evaluated in the phase 2 BEACH301 dose-escalation and dose-expansion study in children aged 3–10 years with achondroplasia and open growth plates (NCT06842355). The study evaluates safety, tolerability, and potentially effective dose levels; clinical efficacy results are not yet available [38].

Approaches that interfere with ligand–receptor interactions have produced contrasting results. Recifercept is a soluble FGFR3 decoy receptor designed to sequester FGF ligands and prevent receptor activation. Although preclinical findings supported this mechanism, the pediatric clinical program was terminated because efficacy was not demonstrated at any of the evaluated doses; the termination was not related to a safety concern. This negative result illustrates the importance of distinguishing biological plausibility from clinically validated therapeutic benefit [39]. By contrast, the anti-FGF2 RNA aptamer RBM-007, now known by the international nonproprietary name umedaptanib pegol, has continued clinical development. An early phase 2 pediatric study has been completed, a long-term phase 2 study has closed recruitment, and a phase 3 pediatric trial evaluating 52 weeks of treatment was registered and began recruitment in 2026. However, peer-reviewed phase 3 efficacy data are not yet available [40,41].

Meclizine represents a drug-repurposing approach intended to attenuate downstream FGFR3–RAS–ERK signaling. Its clinical evidence remains exploratory. In a 26-week, open-label, single-arm phase 2 study, nine children aged 5–10 years received daily meclizine together with growth hormone. Mean height velocity changed only slightly, from 4.35 ± 1.36 cm/year before treatment to 4.46 ± 1.54 cm/year during treatment. Therefore, no clear efficacy for improving linear growth was demonstrated. The reported increase in arm-span velocity remains exploratory, and interpretation is limited by the very small sample size, absence of a control group, short follow-up, and concomitant growth hormone treatment [42]. Another recently initiated program is BMN 333, a long-acting CNP-based therapy designed for once-weekly administration, which is being directly compared with daily vosoritide in the ongoing randomized, active-controlled Phase 2/3 ASPEN trial (NCT07441876). The active-comparator design may provide clinically useful information regarding relative treatment burden and efficacy; however, no efficacy or safety outcomes from the ASPEN trial are yet available, with an update from the dose-finding phase expected in 2027 [43,44].

Research has also begun to identify targets outside the classical FGFR3 pathways. One example is Dkk1 overexpression in mandibular cartilage in the Fgfr3Y367C/+ mouse model. DKK1 inhibits Wnt/β-catenin signaling, and its overexpression appears to reduce canonical Wnt activity in mutant chondrocytes. Inhibition of Dkk1 with WAY262611 increased nuclear β-catenin accumulation, improved mandibular growth ex vivo, increased condylar cartilage volume, and partially corrected abnormal hypertrophic differentiation and chondrocyte proliferation [16]. This suggests that the DKK1/Wnt axis may be particularly relevant for craniofacial manifestations, where current treatment options remain limited. Other experimental approaches are still at an earlier stage. (-)-Epicatechin may modulate downstream FGFR3-related pathways and has improved bone elongation and primary cilium defects in murine models. Teriparatide has produced partial improvement in skeletal development in animal models. Statins have also been investigated, but results are inconsistent: some studies suggest phenotypic benefit, while others do not convincingly show inhibition of FGFR signaling in chondrocytes.

**Figure 2:**
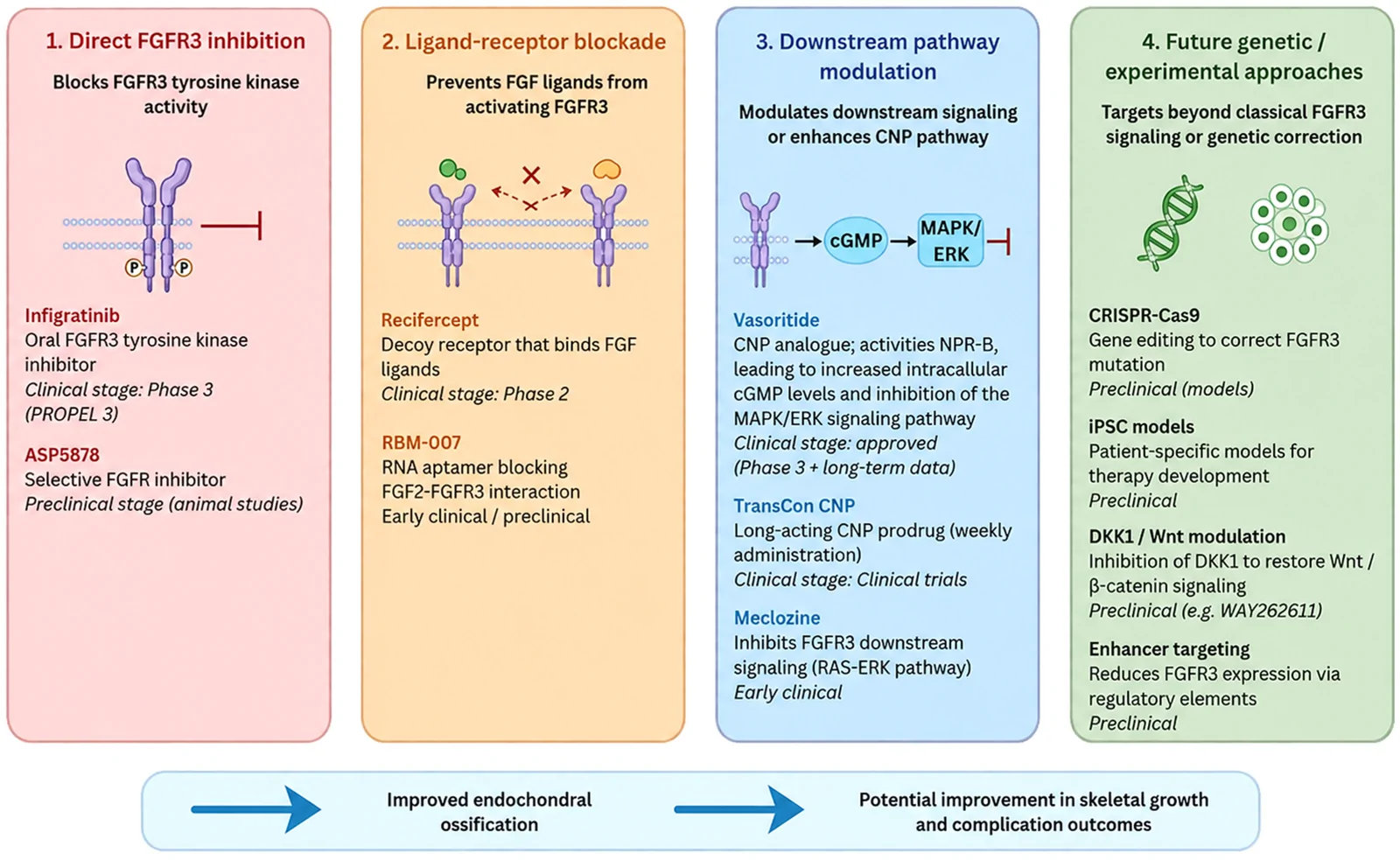
Emerging therapeutic strategies in achondroplasia. Current and investigational therapies target different mechanisms involved in FGFR3 overactivation, including receptor inhibition, modulation of downstream signaling, ligand-receptor blockade, and experimental genetic approaches. The common therapeutic goal is improvement of endochondral ossification and skeletal growth. Created by Biorender.com: https://app.biorender.com/illustrations/canvas-beta/6a5e2c9743ac3f97e1ffb1ff (accessed on 1 June 2026) and adapted from references [9,17,21].

Gene-based strategies remain more speculative, but they are conceptually important. CRISPR-Cas9 approaches and induced pluripotent stem cell models have shown that correction of the FGFR3 mutation in patient-derived cells can restore chondrogenic differentiation [9]. A related strategy is to reduce FGFR3 expression by targeting cartilage-specific enhancer elements rather than correcting the point mutation itself. An additional preclinical RNA-based approach is SIS-102-ACH, a systemic Bio-Courier-formulated siRNA designed to selectively reduce the expression of mutant FGFR3 while preserving the healthy allele. Although it directly targets the molecular cause of achondroplasia, peer-reviewed clinical safety and efficacy data are not yet available. These approaches are not close to routine clinical use, but they expand the therapeutic field beyond receptor inhibition and pathway modulation. Many practical questions remain unanswered. For most emerging therapies, it is not yet clear whether they will improve final adult height, long-term body proportions, limb deformities, cranio-spinal complications, quality of life, or future orthopedic surgery requirements. The same uncertainty applies to combined strategies involving vosoritide and limb surgery. Current expert opinion is cautious: evidence is insufficient either to recommend or reject the combination, and effects on bone healing, callus formation, consolidation, and growth plate behavior remain incompletely understood [11]. Emerging therapies should be viewed as part of a broader shift in achondroplasia care. Treatment is moving away from purely symptomatic management toward modulation of several biological mechanisms involved in skeletal growth. Future care will probably become more individualized, based on age, phenotype severity, complication profile, therapeutic goals, and family preferences. Continuous updating of clinical trial data will be essential as this field evolves (Table 2 and Figure 2) [9,12,16,33].

Combination therapy targeting complementary growth-plate mechanisms has recently emerged as an additional therapeutic strategy. In the phase 2 COACH trial, 21 children aged 2–11 years received once-weekly navepegritide (TransCon CNP) in combination with once-weekly lonapegsomatropin (TransCon hGH). At week 52, treatment-naïve children receiving combination therapy achieved a least-squares mean annualized growth velocity of 8.69 cm/year compared with 5.95 cm/year in matched children receiving navepegritide monotherapy, corresponding to a treatment difference of 2.74 cm/year (*p* < 0.0001). Improvements in body proportionality and arm span were also observed, and treatment was generally well tolerated without treatment discontinuations. Although these findings support the potential value of targeting complementary CNP- and GH-mediated growth mechanisms, they remain preliminary given the small sample size and phase 2 study design, and the long-term effects on final adult height and skeletal complications remain unknown [45].

### 5.4. Comparative Therapeutic Appraisal and Pediatric Outcomes Beyond Linear Growth

Direct ranking of current therapies is not appropriate because available trials differ in participant age, baseline growth assessment, comparator, treatment duration, and outcome definitions. The ongoing ASPEN trial is the first active-controlled comparison of weekly BMN 333 with daily vosoritide, but no results are currently available. Negative or inconclusive programs are also informative: recifercept was terminated because efficacy was not demonstrated, while the small uncontrolled meclizine study did not demonstrate a clear improvement in linear growth. These examples show that biological plausibility does not necessarily translate into clinically meaningful benefit. The clinical value of disease-modifying therapy in pediatric achondroplasia should be assessed using age-specific outcomes. In infants and young children, the most important unanswered question is whether early treatment can favorably modify cranial base development, foramen magnum growth, cervicomedullary compression, sleep-disordered breathing, motor development, or the need for decompression surgery. Although early initiation may be biologically plausible because skeletal growth is most active during the first years of life, current evidence remains insufficient to establish prevention of these complications. Standard neurological, respiratory, and imaging surveillance must therefore continue irrespective of pharmacological treatment.

In older children and adolescents, therapeutic evaluation should incorporate body proportionality, lower-limb alignment, spinal development, pain, mobility, endurance, self-care, school participation, and the requirement for guided growth, osteotomy, or limb-lengthening procedures. An increase in annualized growth velocity cannot automatically be assumed to improve all these outcomes. Moreover, changes in standing height, height z-score, upper-to-lower body segment ratio, arm span, and mechanical axis alignment represent different biological and clinical domains and should be reported separately. Long-term studies should determine whether early pharmacological treatment modifies the natural history of orthopedic and neurological complications rather than merely increasing linear growth during treatment.

Treatment administration is also clinically relevant in children. Daily injections, weekly injections, and oral administration differ in discomfort, treatment fatigue, family workload, adherence, storage requirements, and acceptability. These factors may influence real-world effectiveness even when biological efficacy is similar. Child age, residual growth potential, phenotype severity, comorbidities, previous surgery, family expectations, and treatment preferences should therefore be incorporated into shared decision-making. Direct numerical comparisons between therapies should be avoided when trials differ in participant age, baseline growth assessment, comparator, outcome definition, and duration of follow-up.

A pediatric core outcome framework for achondroplasia trials should include annualized growth velocity and final adult height alongside body proportionality, cranio-cervical and spinal development, limb alignment, orthopedic procedures, respiratory and neurological complications, physical functioning, pain, health-related quality of life, treatment adherence, and child- and caregiver-reported experience. Such an approach would allow future studies to determine whether disease-modifying therapies produce broader health benefits rather than isolated changes in height.

## 6. Future Perspectives

Precision therapies have changed the direction of achondroplasia management by introducing the possibility of modifying parts of the disease course, rather than only treating its consequences. This shift is important, but it should not be overstated. Achondroplasia remains a multisystem condition, and future treatment will still need to be integrated into long-term monitoring, functional support, psychosocial care, and patient advocacy. A major priority is the collection of real-world evidence. As more infants and young children begin treatment early, routine clinical data will become essential for understanding long-term safety and effectiveness outside trial settings. This is especially relevant for children treated in the neonatal period or early infancy, where experience remains limited. Current recommendations will likely need revision as evidence accumulates. One unresolved question is whether treatment can influence structural complications, not only growth velocity. Outcomes such as foramen magnum stenosis, spinal canal development, limb deformities, and axial skeletal growth remain incompletely defined. For cranio-cervical abnormalities, longitudinal MRI studies will be needed to clarify growth patterns, refine screening intervals, and link imaging findings more accurately with clinical decisions.

Future research will also need to move beyond a narrow focus on longitudinal growth and MAPK inhibition. FGFR3 remains central, but other pathways, including PI3K/AKT, Wnt/β-catenin, BMP, hedgehog, and retinoid signaling, may contribute to growth plate biology and skeletal development [7]. Single-cell analyses, multi-omics approaches, and improved animal models may help define the cellular complexity of the disease and identify more precise therapeutic targets. The relationship between pharmacological therapy and orthopedic management is another open area. If emerging treatments alter skeletal growth or deformity progression, they may also change the timing and indications for guided growth, limb lengthening, or corrective osteotomies. At present, there are no universal protocols for combining these approaches. Prospective data and multidisciplinary decision-making will be needed, with patients and families involved in defining realistic goals and acceptable trade-offs.

The combination of vosoritide with orthopedic interventions remains particularly uncertain. Earlier treatment may plausibly improve final height and body proportions and perhaps reduce some complications, but it is not yet clear whether it decreases surgical need, changes deformity recurrence, or affects bone healing after osteotomy [6]. Until stronger evidence is available, decisions should remain individualized according to residual growth, functional priorities, and patient preference. New molecular targets are also beginning to expand the field beyond FGFR3-centered strategies. Dysregulation of Wnt/β-catenin signaling has been implicated in craniofacial development, and DKK1 inhibition has shown promising effects on mandibular growth and chondrocyte differentiation in experimental models. These findings suggest that future treatment may become more region-specific, addressing craniofacial or axial abnormalities as well as long-bone growth.

Gene-based and epigenetic strategies are still speculative, but they may be transformative in the longer term. Experimental work suggests that reducing FGFR3 expression, rather than correcting the mutation itself, can improve skeletal development. Targeting cartilage-specific enhancers such as -29E has produced preclinical improvements in long bone growth, vertebral development, and foramen magnum size. Approaches such as CRISPR-mediated enhancer modulation, RNA interference, or other gene-silencing strategies are promising in concept, but major barriers remain: delivery to avascular cartilage, sufficient targeting of relevant cells, and identification of the safest and most effective timing. Future treatment will also need to address adherence and treatment burden. Experience with vosoritide shows that long-term benefit depends not only on biological efficacy but also on the ability of families and patients to sustain daily injections, monitoring, and follow-up. Because treatment is elective and can be stopped without immediate medical harm, shared decision-making remains central. Families need realistic expectations, clear communication, and ongoing support. The future of achondroplasia care is unlikely to depend on a single definitive therapy. A more plausible model is individualized care combining pharmacological, orthopedic, rehabilitative, and possibly genetic strategies, adapted to age, phenotype, complications, and patient priorities. As the field evolves, evidence will need to be re-evaluated continuously and integrated carefully into clinical practice.

## 7. Conclusions

Achondroplasia is predominantly caused by gain-of-function FGFR3 variants that disrupt growth plate homeostasis and endochondral ossification through interconnected signaling pathways. Vosoritide has demonstrated that FGFR3-related growth plate dysfunction can be pharmacologically modified, while several additional therapeutic strategies are undergoing clinical or preclinical evaluation. Multidisciplinary surveillance remains essential because the long-term effects of disease-modifying therapies on final height, skeletal proportionality, cranio-spinal complications, and orthopedic outcomes remain uncertain.

## Figures and Tables

**Table 1:** Overview of the genetic and molecular basis of achondroplasia.

References	Aspect	Key Findings	Clinical/Pathophysiological Relevance
[4,7,8,9]	FGFR3 structure, expression, and physiological role	FGFR3, located on chromosome 4p16.3, encodes a transmembrane receptor tyrosine kinase expressed predominantly in growth plate chondrocytes, where it acts as a negative regulator of endochondral bone growth.	Links the genetic defect to impaired long-bone, cranial-base, vertebral, and broader skeletal development.
[3,27]	Inheritance pattern	Autosomal dominant inheritance with essentially complete penetrance; around 80% of cases arise de novo.	A single pathogenic allele is sufficient to produce disease, with direct implications for diagnosis and genetic counseling.
[4,5,6,28]	Recurrent mutation	Approximately 99% of individuals carry c.1138G>A (about 98%) or c.1138G>C (about 1%), both resulting in p.Gly380Arg.	Explains the molecular homogeneity of achondroplasia and the relative simplicity of targeted genetic confirmation.
[9,28]	Rare variants	Rare FGFR3 variants include p.Ser217Cys, p.Ser279Cys, p.Ser344Cys, p.Gly375Cys, p.Thr394Ser, and other uncommon substitutions.	Shows that the mutational spectrum is broader than the classic variant, although alternative mutations remain rare.
[7,9]	Gain-of-function receptor activation	Pathogenic FGFR3 variants cause excessive receptor activity through partial ligand independence, increased dimerization and kinase activity, greater receptor stability, and reduced lysosomal degradation.	Defines the central pathogenic mechanism and supports direct or indirect suppression of excessive FGFR3 signaling.
[8,9,15]	Downstream signaling	FGFR3 overactivation affects MAPK/ERK, PI3K/AKT, STAT, RAS/MAPK, and possibly Wnt/β-catenin signaling.	Links the mutation to broad disturbances in chondrocyte proliferation, differentiation, and cartilage homeostasis.
[7,8,9]	Cellular consequences	Excessive FGFR3 signaling inhibits chondrocyte proliferation, suppresses hypertrophic differentiation, and disrupts trabecular bone formation.	Connects molecular dysfunction with defective endochondral ossification and reduced longitudinal bone growth.
[6,9,10,28]	Genotype–phenotype modifiers	The classic phenotype is strongly linked to p.Gly380Arg, but rare variants, compound mutations, and differences in FGFR3 expression level may influence severity.	Explains why achondroplasia is genetically homogeneous in most cases, while still showing biologically meaningful variation.
[10]	Regulation of FGFR3 expression	Cartilage FGFR3 expression is influenced by cis-regulatory elements such as the −29E enhancer; experimental reduction of Fgfr3 expression improved skeletal abnormalities in mouse models of achondroplasia.	Supports FGFR3-expression modulation as a potential therapeutic strategy distinct from direct receptor inhibition.

**Table 2:** Comparative pediatric clinical evidence for current and emerging disease-modifying therapies in achondroplasia [9,10,11,12,14,16,20,33].

Therapy Type	Examples	Mechanism of Action	Administration	Evidence Stage	Main Advantages	Limitations/Uncertainties
Traditional management	Decompression, osteotomy, limb lengthening, growth hormone, rehabilitation	Symptomatic management; does not target the FGFR3 pathway	Surgical/supportive	Established clinical practice	Addresses specific complications and functional needs	Does not modify the underlying molecular mechanism
CNP analogue	Vosoritide	Activates NPR-B/cGMP signaling and attenuates downstream MAPK/ERK activity	Daily subcutaneous injection	Approved; phase 3 and long-term data	First approved disease-modifying therapy	Daily treatment burden; variable response; final-height and complication outcomes remain uncertain
Long-acting CNP analogue	Navepegritide (TransCon CNP; Yuviwel)	Provides sustained CNP signaling and modulates FGFR3-related pathways	Weekly subcutaneous injection	FDA accelerated approval (2026)	Reduced injection frequency	Confirmation of benefit on final adult height remains required
FGFR inhibitor	Infigratinib	Direct inhibition of FGFR1–3 tyrosine kinase activity	Once-daily oral	Phase 3 (PROPEL 3)	Targets the core pathogenic pathway and avoids injections	Investigational; final-height, structural, and long-term safety outcomes remain unknown
FGFR3-selective inhibitor	Dabogratinib (TYRA-300)	Selective inhibition of FGFR3 tyrosine kinase activity	Once-daily oral	Ongoing phase 2 (BEACH301)	Potential first-in-class selective FGFR3 inhibitor with oral administration	No efficacy results yet; long-term safety remains unknown
Decoy receptor	Recifercept	Binds FGF ligands and prevents FGFR3 activation	Injectable	Phase 2 program terminated	Upstream pathway targeting	No efficacy demonstrated at the evaluated doses
RNA aptamer	Umedaptanib pegol (RBM-007)	Blocks the FGF2–FGFR3 interaction	Injectable	Phase 3 ongoing	Specific ligand–receptor inhibition	No peer-reviewed phase 3 efficacy data are currently available
Drug repurposing	Meclizine	Attenuates downstream FGFR3–RAS–ERK signaling	Oral	Exploratory phase 2	Accessible oral agent with a distinct mechanism	No clear linear-growth efficacy demonstrated; evidence is based on a very small uncontrolled study with concomitant growth hormone
Wnt-pathway modulation	DKK1 inhibition (e.g., WAY262611)	Restores Wnt/β-catenin signaling	Experimental	Preclinical	Potential region-specific craniofacial effects	Not clinically available
RNA interference	SIS-102-ACH	Systemic Bio-Courier siRNA designed to inhibit mutant FGFR3 expression	Systemic, investigational	Preclinical development	Mutation-directed FGFR3 silencing	No human safety or efficacy data
Gene- and enhancer-based approaches	CRISPR-Cas9, iPSC models, −29E enhancer targeting	Corrects the FGFR3 mutation or reduces FGFR3 expression	Experimental	Preclinical	Potential for precise and durable molecular intervention	Delivery to cartilage, treatment timing, off-target effects, and clinical translation remain major barriers
Combination therapy	Navepegritide + lonapegsomatropin (TransCon CNP + TransCon hGH)	Complementary CNP-mediated attenuation of FGFR3/MAPK signaling and GH/IGF-1-mediated stimulation of growth-plate activity	Two once-weekly subcutaneous injections	Phase 2 COACH; 52-week data	Greater AGV than navepegritide monotherapy; improvements in body proportionality and arm span	Small, open-label phase 2 study; long-term safety, final-height, and structural outcomes remain unknown

## Data Availability

No new data were created or analyzed in this study. Data sharing is not applicable to this article.
